# Particle Size Related Effects of Multi-Component Flame-Retardant Systems in poly(butadiene terephthalate)

**DOI:** 10.3390/polym12061315

**Published:** 2020-06-09

**Authors:** Florian Tomiak, Bernhard Schartel, Michael Wolf, Dietmar Drummer

**Affiliations:** 1 Institute of Polymer Technology, Friedrich-Alexander-University Erlangen-Nuremberg, Am Weichselgarten 9, 91058 Erlangen, Germany; michael.wolf@fau.de (M.W.); dietmar.drummer@fau.de (D.D.); 2 Bavarian Polymer Institute, Friedrich-Alexander-University Erlangen-Nuremberg, Dr. Mack Strasse 77, 90762 Fürth, Germany; 3 Bundesanstalt für Materialforschung und –Prüfung (BAM), Unter den Eichen 87, 11205 Berlin, Germany; bernhard.schartel@bam.de

**Keywords:** flame retardants, Aluminum diethylphosphinate, boehmite, poly(butadiene terephthalates) (PBT), mechanical properties

## Abstract

Aluminum tris-(diethylphosphinate) (AlPi) is known to have an efficient flame-retardant effect when used in poly(butadiene terephthalates) (PBT). Additionally, better flame-retardant effects can be achieved through the partial substitution of AlPi by boehmite in multi-component systems, which have been shown to be an effective synergist due to cooling effects and residue formation. Although the potential of beneficial effects is generally well known, the influence of particle sizes and behavior in synergistic compositions are still unknown. Within this paper, it is shown that the synergistic effects in flammability measured by limiting oxygen index (LOI) can vary depending on the particle size distribution used in PBT. In conducting thermogravimetric analysis (TGA) measurements, it was observed that smaller boehmite particles result in slightly increased char yields, most probably due to increased reactivity of the metal oxides formed, and they react slightly earlier than larger boehmite particles. This leads to an earlier release of water into the system enhancing the hydrolysis of PBT. Supported by Fourier transformation infrared spectroscopy (FTIR), we propose that the later reactions of the larger boehmite particles decrease the portion of highly flammable tetrahydrofuran in the gas phase within early burning stages. Therefore, the LOI index increased by 4 vol.% when lager boehmite particles were used for the synergistic mixture.

## 1. Introduction

Most polymers are easily flammable under normal atmospheric conditions, promote fire propagation through the release of heat, and release toxic gases during decomposition, due to the chemical structure. Thermoplastic polymers can be modified by admixture of flame-retardant additives in order to reduce the risk of fire, especially in fire-critical applications. Flame retardant additives are classified according to their main active substance, whereby their general efficiency decreases in the following order: halogen-containing, phosphorus-containing, nitrogen-containing, and inorganic [1]. The protective impact of a polymer/flame retardant additive mixture depends not only on the effect of the flame retardant additive itself, but also on the interactions between the polymer, the flame retardant, and further ingredients [2,3,4,5]. Besides the oxygen saturation, chemical reactions are particularly dependent on local temperatures, whereas the temperature profile over time is crucially influenced by heating rates, geometrical, and material properties. Additionally, some studies have shown that by changing the particle size and shape of flame retardant additives, decomposition pathways can vary due to changed thermal conditions and, therefore, affect flame retardant properties [3]. Accordingly, some studies show a delay in the ignition point when the particle size of aluminum hydroxides (ATH) are reduced and mixed in ethylene-vinyl acetate copolymer (EVA) [6] or in polymethyl methacrylate (PMMA) [7]. Other studies reveal a slight increase in the limiting oxygen index (LOI) [8] or denser charring structures when smaller particle sizes are used [9]. Different particle sizes may lead to changes in chemical decomposition products. This was proven for large ATH particles, which decompose in an intermediate stage forming boehmite (AlOOH) instead of a single-stage reaction to aluminum oxide (Al_2_O_3_) and water (H_2_O) for lower particle sizes. The thermally more stable boehmite causes a partial shift in water release towards higher temperatures, having relevant effects on the fire protection properties [4,10]. Accordingly, there is proof that particle size and shape can affect flame retardant performances by changing decomposition pathways, although the topic has been mostly neglected [3].

Flame retardant systems often consist of several components, achieving an improved flame resistance through synergistic effects. These multi-component systems react as compound by different chemical reactions acting serially or simultaneously, whereas the effect that is shown in combination with the matrix polymer is sometimes strongly dependent on the reaction sequence over time or temperature. Accordingly, a high understanding of interactions between all material components is required to ensure good flame-retardant performance at different scenarios. Although numerous synergisms of material pairings are known in the literature, particle size-related influences on flame retardant properties in multi-component systems have not been focus of researchers. Yet, systematic knowledge is necessary to describe particle size related changes in decomposition pathways in order to understand their influence on flame retarding properties.

Aluminum tris-(diethylphosphinate) (AlPi) has generally been proven in multiple studies to be efficient flame inhibitors for both reinforced [11,12] and neat polyesters [13,14,15]. They act through the formation of a carbon layer as well as through the chemical neutralization of radicals in the gas phase. In specific proportions, metal hydrates, boehmite, and metal oxides can be used as synergist in combination with AlPi [16,17], acting through an endothermic release of water and the formation of an oxide layer [18]. Beneficial interactions between a phosphorous flame retardant and the right concentration and size of boehmite were also reported for other phosphorus flame retardants [19,20]. A commercially available AlPi and two differently sized boehmite types (B60, 200SM) were mixed with poly(butadiene therepthalate) (PBT) to identify the synergistic effect as a function of the particle size distribution. LOI and UL-94 fire testing were performed in order to identify synergistic effects and characterize the differences in fire inhibiting properties between the particles’ sizes used. Thermogravimetrical analysis (TGA) in combination with Fourier transformation infrared spectroscopy (FTIR) as well as scanning electron microscopy (SEM) were used to study the decomposition processes, leading to different results in fire testing. The aim of the paper is to identify differences in fire behavior that are caused by the use of differently sized particles in multi-component flame retardant systems. Here, it shall be worked out to what extent particle sizes can influence the thermal decomposition process at different heating rates and, thus, change the interaction of all components within the flame-retardant system. Possible causes for changed flame retardancy mechanisms are discussed and explanations proposed.

## 2. Materials and Methods

### 2.1. Materials and Sample Preparation

As matrix polymer, a standard PBT grade Ultradur B 4520 supplied by BASF SE (Ludwigshafen, Germany) was used for the investigation. The flame-retardant effect was gained by defined compositions of an AlPi (Exolit 1240_d90_ < 77 μm, density 2.5 g/cm³) provided by Clariant AG (Muttenz, Switzerland) and two different boehmite grades (ACTILOX^®^ B60, ACTILOX^®^ 200SM) provided by Nabaltec AG (Schwandorf, Germany) as synergist [21]. The boehmite grades B60 and 200SM are chemically identical, but they differ in particle size (B60_d90_ < 5 μm, 200SM_d90_ < 0.6 μm) and specific surface area (BET, B60_d90_ ≈ 5 m^2^/g, 200SM_d90_ ≈ 17 m^2^/g) [22]. The material components were prepared using a twin-screw extruder DSE ZSE HP 27 from Leistritz GmbH (Nuremberg, Germany) with controlled temperatures between die and granulate feedblock of 260/255/255/250/250/245 °C. Specific compositions of AlPi and boehmite were premixed using a universal powder mixer. Table 1 shows all material compositions. The polymer strand was drawn off via a water bath, chipped and dried afterwards. 1:1 standard campus tensile bars (type A) with a length of 170 mm and a thickness of 4 mm were used as test specimens and produced by injection molding. The tensile bars were produced using an Arburg 370 Allrounder from Arburg GmbH (Loßburg, Germany). For LOI and UL-94 testing 80 mm specimens with edge length of 10 and 4 mm thickness have been prepared from the tensile area of the tensile bars by sawing and milling.

### 2.2. Characterization and Testing

#### 2.2.1. Fire Testing

LOI (limiting oxygen index or oxygen index (OI), DIN EN ISO 4589), and UL-94 (DIN EN 60695) tests were conducted to determine the flammability for all of the materials used. The fire tests were carried out using an oxygen index analyzer and an UL-94 fire-testing device from Taurus Instruments AG (Weimar, Germany). Test flames were calibrated to 50 W. All of the tests were carried out in accordance to standards. LOI is widely used to characterize polymer materials and indicates the minimal atmospheric oxygen content that is required to sustain a combustion process in a candle-like set-up visible as a flame. The vertical UL-94 fire test was used to describe the ability to self-extinguish once ignited under normal atmospheric oxygen. The test results are then described in three classifications V-0, V-1, V-2, whereas V-0 represents an almost instant self-extinguishing with or without non-burn-dripping behavior. V-1 classifications indicate slightly longer self-extinguishing times, whereas V-2 classifications show self-extinguishing behavior accompanied by burn dripping. 

#### 2.2.2. Microscopy

Scanning electron microscope (SEM) studies were performed to identify the overall particle shape. The powder samples were spattered with a platinum-palladium mixture. A SEM Ultra Plus system from Zeiss AG (Oberkochen, Germany) was used for the analysis. The SEM characterization of fire residues were done using an EVO A10 (Zeiss, Jena, Germany) with an acceleration voltage of 10 kV. The fire residues were produced with 20 mm × 20 mm × 4 mm specimen burnt under the cone heater of a cone calorimeter from Fire Testing Technology Ltd. (FTT) (East Grinstead, UK) applying 50 kW/m^2^. The surface, the inner structure, and the cross-section based on a side view were characterized of the 15 nm gold-sputtered fire residues.

#### 2.2.3. Thermal Analysis and Evolved Gas Analysis

Thermogravimetrical analysis (TGA) was carried out to identify changes in the decomposition process that is triggered through individual flame-retardant compositions used. Measurements were performed using a STA 449 F3 Jupiter from Netzsch GmbH (Selb, Germany) and conducted at an overall heating rate of 20 K/min. Additional higher rates of 200 and 500 K/min were applied to boehmite powders and PBT containing 20 wt.% AlPi (A4) to identify particle size related shifts in reaction profiles considering realistic heating rates given in fires at the specimen surface. Detected relative shifts of the reaction profiles are displayed and compared. Additionally, TGA measurements coupled with Fourier transformation infrared spectroscopy (FTIR) were carried out to analyze pyrolysis gases of PBT containing 20 wt.% AlPi (A4) as well as PBT containing 15 wt.% AlPi and 5 wt.% B60/200SM boehmite (B1, C1). For TGA measurements with coupled evolved gas analysis, a FTIR device Tensor 2 from Bruker Corp. (Billerica, Massachusetts, USA) was used. Thus, changed decomposition pathways related to differently sized boehmite particles were investigated. All of the transfer parts (adapters and transfer line) were temperature controlled at 230 °C, the FTIR gas cell at 200 °C. The tests were carried out at a heating rate of 5 K/min. to ensure high spectral resolutions. The spectra were averaged with 32 scans each. Each averaged spectrum corresponds to a temperature window of about 2.5 K (30 s per spectrum). All of the tests were performed under both oxygen and nitrogen atmosphere with a defined gas flowrate of 70 mL. Specimen weights are controlled between 8 and 10 mg. Table 2 lists all TGA and TGA-FTIR tests performed.

#### 2.2.4. Mechanical Testing

Standard tensile tests (DIN EN ISO 527) and impact bending tests (DIN EN ISO 179-1) were performed for all material mixtures to analyze changes regarding the mechanical performance. Therefore, a universal tensile test rig from Zwick & Roell GmbH (Ulm, Germany) was used. The traverse speed was defined at 50 mm/s. The clamping length was 115 mm, the preload force 1 N at 1 mm/min. tensile speed. The impact bending test was carried out according to the standard DIN EN ISO 179-1/1eU. The samples without notch of type 1 with the dimensions 80 mm × 10 mm × 4 mm were tested by an impact on the narrow side. Nine specimens per material were tested. A 15 J pendulum was used for pure PBT since it is known that PBT is relatively impact-resistant. A 7.5 J pendulum was used for the materials modified with flame retardant additives. All tests were performed under norm climate.

## 3. Results

### 3.1. Microscopy—Particle Size and Particle Distribution

**Figure 1:**
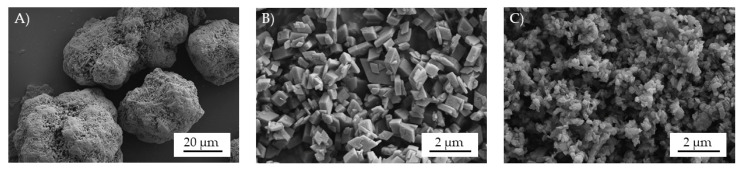
SEM images of the used flame retardant powder particels, (**A**) AlPi (Exolit OP 1240), (**B**) boehmite powder Actilox B60, and (**C**) boehmite powder Actilox 200SM.

Figure 1 shows SEM images of the flame retardant powder particles of AlPi (Exolit OP 1240) and boehmite (Actilox B60; Actilox 200SM). The AlPi particles are largely spherical with a rough surface and a mean particle diameter being given by the manufacturer of about 41 µm as well as a specific surface area of 1.2 m²/g [21]. In contrast, the boehmite particles are angular and tend to strongly agglomerate. The SEM results confirmed that the boehmite grades Actilox B60 and 200SM have an average particle diameter of 1.2 and 0.35 µm and, thus, are about 30–120 times smaller than the AlPi particles [22]. Accordingly, key figures for the specific surface area of B60 and 200SM are specified with 5 and 17 m²/g [22]. 

**Figure 2:**
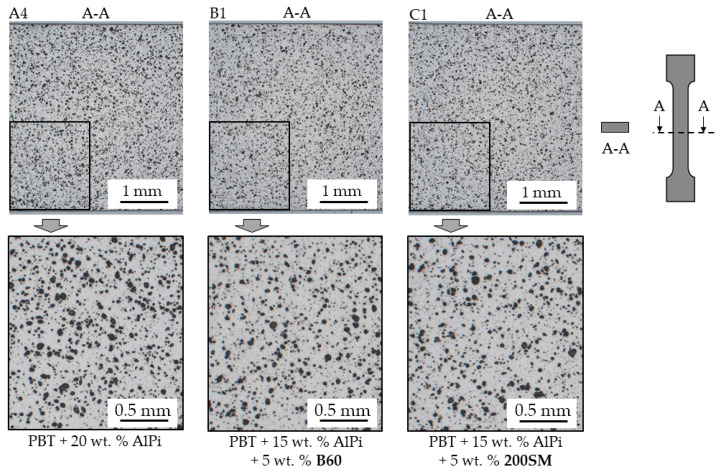
Selection of light microscopy images of the tension rod cross section (A-A) taken in central tension area. Homogeneous particle distribution and no visible agglomerations.

Specimens were also examined by light microscopy to identify possible process-caused inhomogeneities (Figure 2). The specimens were prepared from the central part of the tensile area (illustrated in Figure 2, right), embedded, polished, and examined. These show a homogeneous distribution over the cross-section without agglomerations or defects. Multi-component mixtures containing AlPi and boehmite were premixed 15–20 min using a powder mixer from Somakon UG (Lünen, Germany) with a rotational speed of 100 rpm before processing. Accordingly, good homogenization is considered.

#### 3.1.1. Mechanical Testing

**Figure 3:**
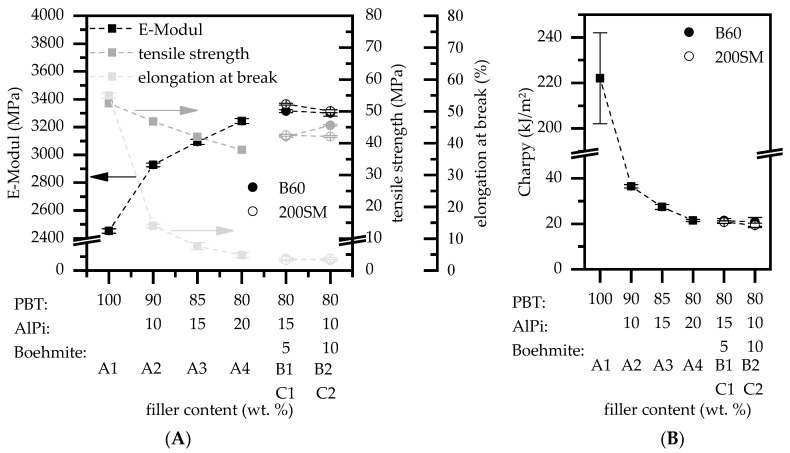
Mechanical properties of flame retardant modified poly(butadiene therepthalate) (PBT) filled at different filler contents of Aluminum tris-(diethylphosphinate) (AlPi) and boehmite mixtures. (**A**) Key figures of tensile tests and (**B**) impact bending tests.

Mechanical properties were investigated by standard tensile and impact bending tests (Figure 3). For increasing AlPi content in PBT/ AlPi mixtures (10–20 wt.%), E-Module indicates higher material strength, whereas tensile strength, elongation at break, and Charpy impact strength decrease significantly (Figure 3). Accordingly, the E-Module for pure PBT and PBT containing 20 wt.% AlPi steadily increases from 2453 to 3243 MPa (∆ +32%), while the tensile strength decreases with a linear character from 53 to 38 MPa (∆ −28%). In contrast, key figures elongation at break as well as charpy impact strength drop rapidly when 10 wt.% AlPi is added into the mixture from 55% and 222 kJ/m² (net PBT) to 14% and 37 kJ/m². Higher filler contents of 15 and 20 wt.% AlPi cause relatively minor changes for elongation at break measured with 8% and 5% as well as charpy impact strength measured with 27 and 21 kJ/m². Therefore, we conclude that results for elongation at break and Charpy impact strength are largely dependent on the overall filler content, whereas a dependency on particle size cannot be determined for the tested material mixtures. It is suggested that the increasing presence of flame retardant additives in the matrix predominantly prevents flow movements under load to relieve stress within the polymer morphology. As a result, local stress peaks occur, leading to brittle fracture behavior. However, the E-Module and tensile strength slightly increase when AlPi is partially substituted by boehmite particles. Here, mixtures containing 5 and 10 wt.% boehmite combined with 15 and 10 wt.% AlPi (B1, B2, C1, C2) show slightly higher values for the E-module and tensile strength than comparable PBT containing 20 wt.% AlPi (A4). We conclude this effect to a finer distribution of the smaller boehmite particles, whose higher surface-to-volume ratio permits a larger surface area and, thus, better polymer-particle bonding. Accordingly, AlPi boehmite mixtures in PBT offer the possibility of achieving higher component strengths when compared to PBT/AlPi mixtures.

#### 3.1.2. Fire Testing

**Figure 4:**
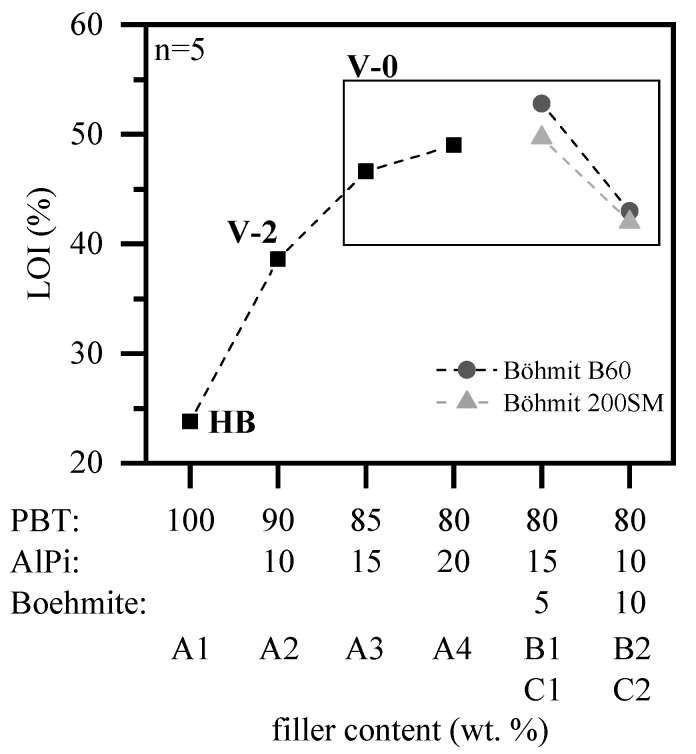
Oxygen index and UL-94 classification of AlPi and boehmite modified PBT.

A standard PBT grade was systematically modified by defined loading levels of AlPi and boehmite in order to improve the flame-retardant properties (Table 1). LOI and UL-94 test results show a subsequent improvement with increasing filler content for all modified test compounds when compared to pure PBT (Figure 4). Accordingly, the oxygen index (OI) increases with the addition of 10, 15, and 20 wt.% AlPi (A1–A4), achieving OI values of 38.6%, 46.6%, and 49.0%. Despite the high OI when 10 wt.% AlPi are added, owing to burn dripping only V-2 classification (UL-94) being achieved. For higher filling degrees of 15 and 20 wt.%, melt dripping occurred non-flaming, so that the UL-94 classification increased to V-0 for both mixtures (A3, A4). 

The flame retardancy effect of AlPi is strongly non-linear, whereas higher filling degrees tend to level off achievable OI values relative to the filling degree. This indicates a decrease in efficiently of chemical reactions, lowering the flame retarding performance at the given PBT/ AlPi ratios. For filling degrees exceeding 20 wt.% AFlPi, we assume a slight further increase of the OI value, leading to a negative flame retardant effect after reaching a turning point. Similar results were reported in other studies while using comparable PBT/AlPi systems [12].

The non-linear dependency of the flame retardancy on the filler concentration pronounces the opportunity of a partial AlPi-replacement by synergistical components, targeting an optimized multi-component flame retardant system [23,24]. Based on a global filler content of 20 wt.% AlPi (A4), the phosphorus main active ingredient was therefore gradually substituted by 5 and 10 wt.% (B1, B2, C1, C2) of two different particle sized boehmite grades B60 (d90 < 5 μm) and 200SM (d90 < 0.6 μm). The results for a loading level of 5 wt.% boehmite and 15 wt.% AlPi (C1) show synergistic effects regarding the oxygen index for both boehmite grades. In particular, larger boehmite particles (B60) seem to achieve a higher OI value of 53% (B1, C1), while material compositions containing identical loadings but the finer boehmite grade 200SM (B1, C1), show comparable values (50%) as PBT containing 20 wt.% AlPi (A4) (49%). Following the curve for PBT/ AlPi compositions, it can be further expected that, even with filling degrees exceeding 20 wt.% AlPi, measured OI values may not reach those measured for PBT/AlPi/boehmite B60 mixtures (B1, C1). All of the mixtures achieved V-0 classification according to UL-94. Higher degrees of substitution with boehmite proportions of 10 wt.% (B2, C2) show a clear reduction of the OI as compared to PBT containing 20 wt.% AlPi (A4), whereby differences between both boehmite types B60 and 200SM are negligible (43% and 42%). Here, V-0 classifications are also reached resulting from non-burning dripping and quick self-extinguishing. For the mixing ratios selected within this study, material mixtures of 15 wt.% AlPi and 5 wt.% boehmite (B1, C1), thus achieving the best flame-retardant effect with visible synergistic improvements compared to PBT mixtures containing 20 wt.% AlPi (A4). The measured deviation of the OI value as a function of the boehmite particle sizes used is investigated in the following chapters. 

#### 3.1.3. Thermal Analysis

**Figure 5:**
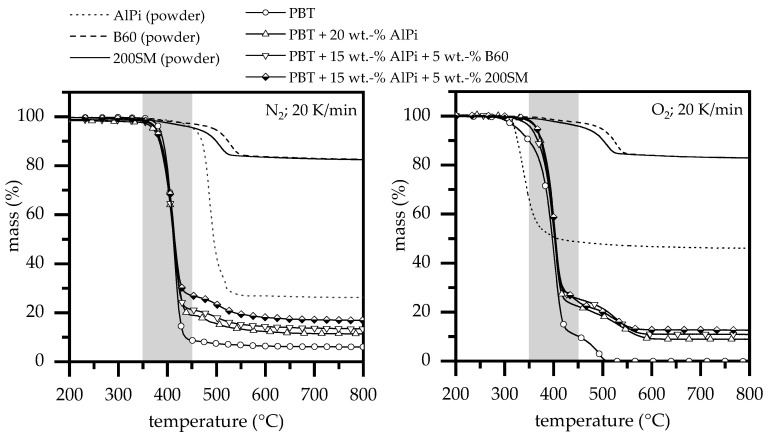
Thermogravimetric analysis (TGA) of all net materials and mixtures measured at a heating rate of 20 K/min. under nitrogen and oxygen atmosphere.

The TGA measurements of pure PBT and AlPi show a clear dependence of the test atmosphere on the decomposition process (Figure 5). For pure PBT, the decomposition onset temperature, which is defined here as 5 wt.% mass loss, shifts from *T*_95; PBT(N)_: 380 °C (A1) under nitrogen to *T*_95; PBT(O)_: 325 °C (A1) under oxygen atmosphere, whereas the temperature difference measured decreases reaching a weight loss of 50 wt.% to *T*_50; PBT(N)_: 412 °C (A1) and *T*_50; PBT(O)_: 397 °C (A1) (Table 3). For TGA measurements under oxygen atmosphere, a second decomposition step between 425 and 500 °C is revealed, forming a temporal shoulder. By reaching 450 °C, the residue rapidly decreases to almost 0% under oxygen atmosphere, marking strong oxidation processes, whereas, under nitrogen atmosphere, only low further degradation can be identified. For net AlPi, the test atmosphere applied seems to have an even greater effect. The decomposition onset temperature shows a shift from *T*_95; AlPi(N)_: 452 °C to *T*_95; AlPi(O)_: 325 °C (∆*T* = 127 °C), whereby the residue almost doubles from about 26 to 46 wt.%. The major difference of AlPi degrading in nitrogen and oxygen atmosphere is not a change in chemical processes, but rather temperature dependent decomposition processes, which occur, according to Samyn [25]. The presence of oxygen shifts the decomposition onset temperature to lower values, whereas phosphinates react to aluminophosphates and phosponates in the condensed phase, leading to no vaporization and high char residues [25,26].

The decomposition of boehmite occurs independently of the test atmosphere by an endothermic reaction to aluminum oxide (Al_2_O_3_) and water [18]. Hereby, TGA measurements reveal shifts in the decomposition process, depending on the particle size used. Smaller particles have larger surface areas as well as higher surface-volume ratios, allowing to decompose more quickly when appropriate temperatures are reached [27]. For the used boehmite particles, the surface area of the 200SM type is almost three times larger (17 m^2^/g) as compared to B60 type particles (5 m^2^/g), leading to a shift of the decomposition onset to lower temperatures. Accordingly, the onset temperature for 200SM is identified at *T*_95; 200SM_: 460 °C, whereas B60 starts at *T*_95; B60_: 495 °C (Table 3). 

The thermal decomposition process of flame retardant PBT mixtures containing solely AlPi (A4) and AlPi/boehmite mixtures (B1, C1) occurs in two gravimetrical steps (Figure 5). The beginning of thermal decomposition is shifted by 10°C to lower temperatures for the flame retarded PBT materials. The char yield increased by 5 wt.% for adding 20 wt.% AlPi, and by 7–10 wt.% for adding 5 wt.% boehmite. The results indicated that adding AlPi mainly results in the release of phosphorus and its flame retardancy activity in the condensed phase is limited. The char yield, when adding boehmite, is formed due to the formation of alumina and its effect as catalytic sites increasing the char residue during PBT decomposition. The slightly higher char yield observed for 200SM is explained well by the higher activity of the formed metal oxides due to their larger surface area. Different effects tend to shift the decomposition process towards lower temperatures when an oxygen rich test atmosphere is applied, as already described earlier for PBT and AlPi. This effect, although significantly lower than measured for net PBT and AlPi, can also be identified for all flame retardant PBT types (Table 3). Accordingly, the decomposition onset temperature under oxygen is about 5–15 °C below the value determined for measurements under nitrogen. While PBT mixtures containing 20 wt.% AlPi (A4) as well as 20 wt.% AlPi/B60 mixtures (B1) measured under oxygen atmosphere show a largely identical decomposition onset temperature of *T*_95_: 364–365 °C, PBT containing 20 wt.% AlPi/200SM mixtures (C1) seem to decompose earlier at *T*_95_: 354 °C. This effect might be caused by an earlier endothermic release of water identified by TGA measurements for smaller boehmite particles (200SM). Since the decomposition of boehmite is independent of the ambient gas atmosphere and cannot be detected in nitrogen atmosphere, it is further assumed that a simultaneous breakdown of the polymeric material triggered through oxidation reactions enhances the effect. This earlier decomposition fits well to LOI testing conditions and might explain the lower oxygen index measured for PBT mixtures containing 5 wt.% of the smaller 200SM boehmite particles (C1). Corresponding aspects are further investigated by FTIR gas analysis in the following chapter. Whereas, the char yield of PBT equals 0 at high temperatures, the char yields of the flame retarded PBT materials were observed to be very similar for the thermo-oxidation, as described for the thermal decomposition. Thus, the increase in residue is even more pronounced. 

#### 3.1.4. Gas Analysis

Pyrolysis gases of PBT containing AlPi and B60/ 200SM boehmite are investigated by TGA-FTIR analysis to investigate changed decomposition pathways, which may explain particle size related differences in LOI and residue values. The analysis was carried both under nitrogen and oxygen atmosphere, while using a heating rate of 5 K/min. For the sake of clarity, only results regarding synergistic mixtures PBT + 15 wt.% AlPi and 5 wt.% boehmite (B60, 200SM) are presented. The analysis showed a gradual decomposition process, whereas the main gas components were identified, as follows: 1,3 butadiene, terephthalic ester, benzoic acid, carbon monoxide (CO), carbon dioxide (CO_2_), tetrahydrofuran (THF), 1,4-butanediol, diethylphosphinic acid, and butanediol. Terephthalic acid mostly condensed in the transfer line due to a high sublimation point at 402°C and could, therefore, not be found in the gas phase [28]. An overview with the characteristic wavelengths is given in Table 4. All of the identified decomposition products correspond well to literature values. Furthermore, the decomposition process of PBT as well as PBT containing AlPi is well known and will therefore not be discussed in detail. Detailed investigations can be found for example in the following studies: [11,12,29,30,31].

**Figure 6:**
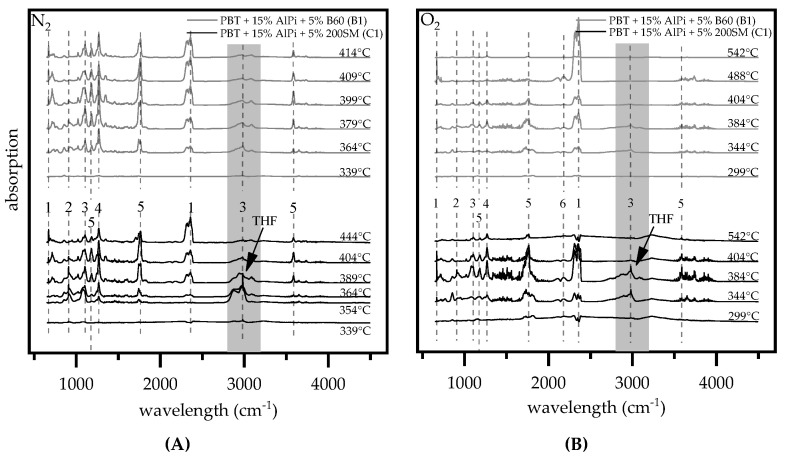
FTIR spectra of evoluted gases conducted under nitrogen (**A**) and oxygen (**B**) atmosphere at a TGA heating rate of 5 K/min.; (**1**) CO_2_, (**2**) 1,3 butadiene, (**3**) THF, (**4**) therephthalic ester, and (**5**) benzoic acid. Spectra indicate changed decomposition paths when different boehmite particel sizes are used as flame retardant in PBT compositions; In particular a significantly higher THF (2978 cm^−1^) formation for smaller boehmite particles can be identified.

Figure 6 shows the result of the FTIR gas phase spectra of the decomposition process for PBT + AlPi/boehmite (200SM, B60) mixtures at selected temperatures and defined atmospheres. Under nitrogen and oxygen atmosphere gas phase products identified at early decomposition stages show the formation of tetrahydrofuran (2978 and 1083 cm^−1^), esters (1743, 1267 and 1100 cm^−1^), and CO_2_ (2450–2300 and 668 cm^−1^). With further decomposition progress at higher temperatures, the proportion of tetrahydrofuran found in the gas phase drops, whereas CO_2_, ester, benzoic acid (3578, 1758 and 1178 cm^−1^) and butadiene (908 cm^−1^) evolve more dominant. Additionally, under oxygen atmosphere, low proportions of CO (2110 and 2181 cm^−1^) are evolved. Fragments of diethylphosphinate can also be found at 850 and 1018 cm^−1^ due to the presence of AlPi within the material mixture. 1,4-butanediol is suspected at characteristic wavelengths of 2982, 2987, and 1072 cm^−1^ [31] but was not clearly detected due to overlapping signals with tetrahydrofuran. 

In accordance with the literature [30,32], PBT mixtures containing AlPi and water releasing minerals promote the formation of tetrahydrofuran found in the gas phase. When comparing PBT + AlPi/boehmite mixtures (B1, C1) in early decomposition stages, a clear difference in the formation of tetrahydrofuran is indicated for measurements under nitrogen and oxygen atmosphere (Figure 6). For PBT mixtures containing the smaller boehmite particles (200SM), the absorption intensities show a higher overall presence of tetrahydrofuran in early decomposition stages and a lower presence of benzoic acid as well as CO_2_ than recorded for mixtures containing larger boehmite particles (B60). Thus, a change in decomposition pathways is suggested. 

In particular, the formation of tetrahydrofuran within the decomposition process of PBT is known to take place at lower temperatures [32]. Two possible thermal reaction paths are described in the literature [30,32]: the initial reaction is caused by chain cleavage of pure PBT via a six-membered cyclic transition state to form benzoic acid and an ester derivative, which in turn reacts to terephthalic acid and butadiene. As the acyl-oxygen cleavage in the ester bonds progresses, intra- or intermolecular H-shifts tent to occur, changing the reaction path to form tetrahydrofuran and the carboxylic acid terminated chain. As a second possibility also caused by H-shifts within the ester group diols can be formed, which convert to tetrahydrofuran (–H_2_O) and butadiene (–2H_2_O) under the elimination of water. Apart from thermal decomposition, the hydrolysis of PBT occurs, not only based on the water solved in PBT, but also due to the water that formed during the decomposition of PBT [16,28]. The release of water by boehmite at the beginning of pyrolysis enhances the hydrolysis and, thus, the formation of THF. The contribution of hydrolysis to the decomposition of PBT is reported to increase with decreasing decomposition temperature [33]. We conclude that the release of water by boehmite evolved the hydrolysis of PBT from a minor to one of the main decomposition pathways in the beginning of decomposition.

**Figure 7:**
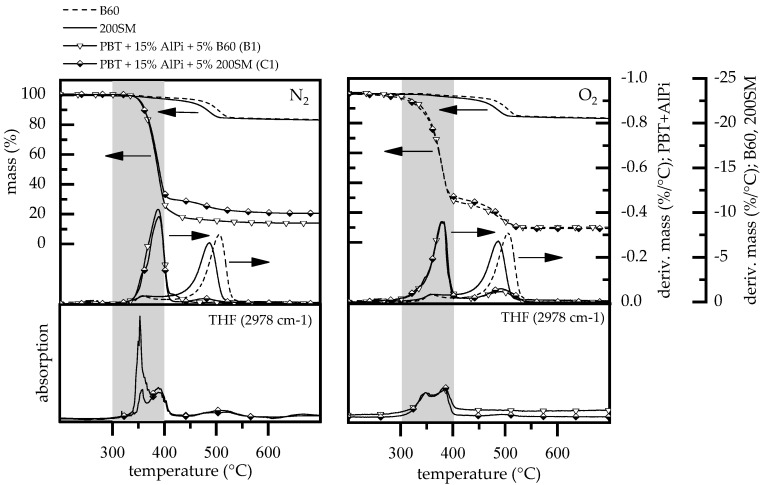
Comparison of TGA curves of pure boehmite, PBT + 15 wt.% AlPi/5 wt.% boehmite mixtures and FTIR spectra for tetrahydrofuran (THF) over time. Heating rate conducted: 5 K/min.

The characteristic wavelength of THF at 2978 cm^−1^ was extracted for pure boehmite (B60, 200SM) and PBT + AlPi/ boehmite mixtures (B1, C1) and compared by TGA measurements (Figure 7) in order to compare the evolution of tetrahydrofuran over time. The decomposition process of boehmite and, therefore, the release of water is shifted to higher temperatures when larger particles are used, as already described earlier. Accordingly, by the time the peak of THF development is reached, smaller (200SM) in comparison with larger (B60) boehmite particles, the quantity of water released into the system is about 40% higher. A much more pronounce enhancement of hydrolysis occurred.

THF are highly flammable gases. Therefore, the early release into the gas phase for mixtures containing smaller 200SM boehmite particles explain the deviating results in LOI testing. 

#### 3.1.5. Heating Rates

**Figure 8:**
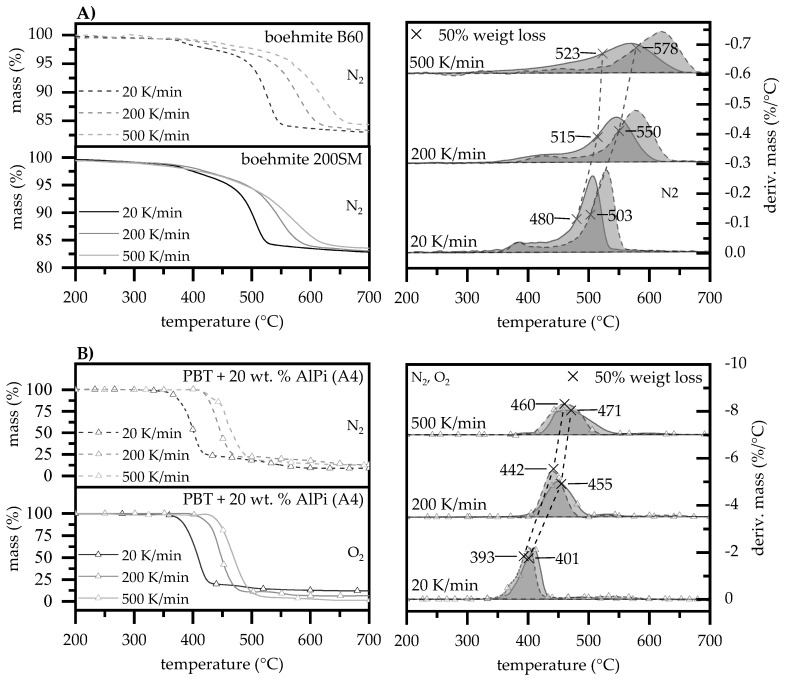
TGA measurements at heating rates 20, 200 and 500 K/min under nitrogen and oxygen atmosphere. Temperature at 50% weight loss measured by total mass loss are marked and connected by a dotted line for (**A**) pure boehmite (**B**) PBT modified by 20 wt.% AlPi.

Boehmite when used as synergists in PBT/AlPi mixtures can influence the decomposition pathway in early decomposition stages, as described earlier. The increase in hydrolysis is proposed to increase the evolution of THFs, thus, as a result, reducing the oxygen index measured. In real fires, materials’ surface exposed to an ignition or heat source experience significantly higher heating rates (10–100 K/s) than those typically used for thermal analyses [34]. Pure boehmite B60 and 200SM as well as PBT containing 20 wt.% AlPi were analyzed at 20, 200, and 500 K/min. via thermogravimetric analysis and compared by weight loss over time and temperature (Figure 8) in order to identify relative changes in decomposition profiles for higher heating rates, which might further influence reaction pathways.

The TGA results show a systematic shift of the thermal decomposition with increasing heating rates towards higher temperatures (Figure 8A). The thermal shift is visibly reduced with increasing heating rates showing a regressive character. The inertial effects of different particle-sized boehmite B60 and 200SM, as described earlier, cause a time-lagged thermal decomposition and, thus, a time-differentiated release of water. As expected, higher heating rates of 200 and 500 K/min. also appear to have a different effect on water release, depending on the particle sizes present. Points were marked relating to a 50% weight loss, as measured by the total release of water, in order to compare the temporal water release. The measurements clearly show an unequal shift in water release at higher heating rates between 20 and 500 K/min. for B60 and 200SM particles from 503 to 578 °C and 480 to 523 °C, respectively. This corresponds to a delta of 23 and 53 °C, marking an increasing offset in water release when higher heating rates are applied. Accordingly, smaller 200SM as compared to larger B60 boehmite particles show a lower dependency of the heating rate applied.

The TGA measurements for PBT/AlPi compounds with increased heating rates also show a shift in the thermal decomposition range towards higher temperatures (Figure 8B). Measurements were carried out under both oxygen and nitrogen atmosphere due to the O_2_ sensitivity of PBT as well as highly oxygen enriched atmospheres within LOI tests. Measured by the gravimetric decomposition peak of the derived mass change, the peaks of thermal decomposition at applied heating rates of 20 and 500 K/min. shift from 292 (O_2_) and 401 °C (N_2_) to 460 (O_2_) and 471 °C (N_2_). The proportion of atmospheric causes only causes a uniform shift of 9 to 13 °C, which only implies minor changes in the decomposition process if different gas compositions are applied.

**Figure 9:**
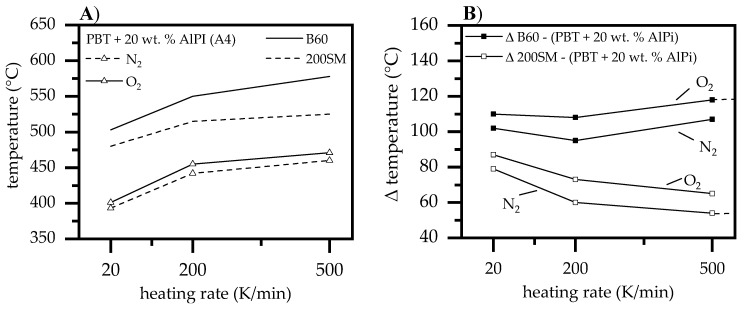
(**A**) Peak values of boehmite particles released 50% water as measured by the maximal and peak temperature of the derived mass change of PBT containing 20 wt.% AlPi (**B**) temperatures boehmite particles released 50% water as measured by the maximal release of water and subtracted from the gravimetric decomposition peak temperature of the derived mass change of PBT containing 20 wt.% AlPi.

Previously determined characteristic peak-temperatures, indicating a mass loss of 50% at different heating rates (cf. Figure 8), were plotted in Figure 9A in order to obtain a direct comparison. Additionally, we subtracted the peak-temperature (mass loss of 50 %) of boehmite B60 and 200SM from the peak-temperature of PBT + 20 wt.% AlPi to visualize relative changes in decomposition processes between PBT containing 20 wt.% AlPi and net boehmite (Figure 9B). 

The characteristic decomposition temperatures increase when higher heating rates are applied showing a regressive curve progression, as mentioned earlier. While the PBT/AlPi mixture (A4) indicate a gas atmosphere independent shift of the decomposition peak, B60 and 200SM boehmite particle seem to behave differently with increasing heating rates applied. Looking at relative temperatures, the behavior becomes clearly visible (Figure 9B). Smaller 200SM boehmite particles tend to decompose relatively earlier than the PBT/AlPi mixture with increasing heating rate. This implies an equally earlier water release in multi-component PBT/AlPi/boehmite mixtures. Consequently, higher water concentrations in earlier decomposition stages of PBT/AlPi mixtures is present, which accelerated the initiation of the previously described formation of THFs by hydrolysis. On the other hand, larger boehmite particles result in a relative delay of water release when higher heating rates are applied, which are caused by an increased inertia effect. Consequently, in multi-component PBT/AlPi/boehmite mixtures, a relative delay in water release is suspected, leading to less water being available in early decomposition stages, thus the evaporation THF is less dominant. Therefore, in summary, it is assumed that the relative shift in decomposition profiles with increasing heating rates favors the release of highly flammable THFs by earlier water release of the smaller boehmite particles.

#### 3.1.6. Residue Structure

**Figure 10:**
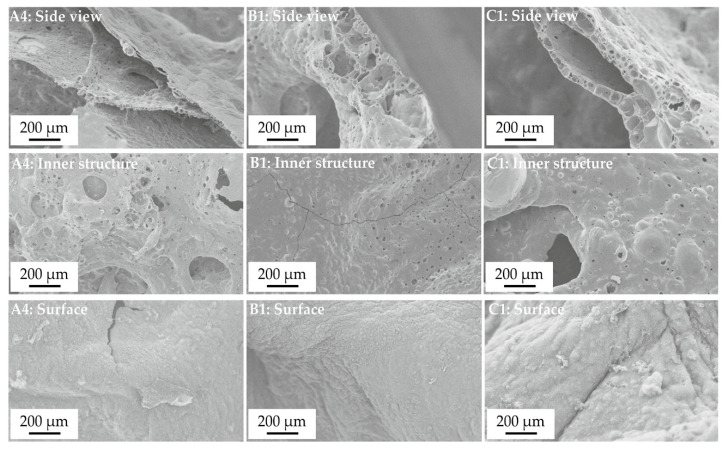
Morphology of the fire residues of **A4**, **B1**, and **C1**. Scanning electron microscope (SEM): side view to get a cross-section of the top of the residue, inner structure and surface top view.

Figure 10 show representative SEM images from the fire residues of A4, B1, and C1. Images were taken from the top of the residue as side view to obtain a cross section from the structure, from the inner part of the residue and a top view on the surface. Adding AlPi to PBT in A4 resulted in a layered residue. As concluded from the TGA and evolved gas analysis, most of the organo-phosphorus of AlPi was released, aluminum phosphate built together with carbon the layered residue. The surface layer looks like only a few-micrometer thin skin with a closed structure, the layers inside the residue were around 10–30 micrometer thick consisting of bubbles and large holes. When some of the AlPi was replaced with boehmite in B1 and C1, fire residue changed visibly across the cross section. The surface layer became foam-like and much thicker, up to several hundreds of micrometers. The inner structures show less holes and more intact large bubbles. Most probably also the inner layers increased in thickness. The top view at the surface did not prove any reliable difference. 

The morphology of the fire residue proves that much more than an increase in residue occurred. In all fire residues, layered structures could be identified, whereas a closed char-surface was most probably favored by aluminum phosphates. Replacing AlPi with boehmite enhances the foam character of the layers and the dramatic increase in thickness by two orders of magnitude. Layered structures, foam-like, and closed surfaces are the properties that characterize fire residues showing pronounced protective layer effects. Thus, apart from increasing the amount of residue and reducing the total amount of fuel released, a protective layer effect is concluded as major flame retardancy mode of action reducing the mass loss rates and, consequently, the heat release rate (HRR). The combination of flame retardant modes of action in the gas phase, flame inhibition due to AlPi, and in the condensed phase, increased charring and protective layer formation provides synergistic flame retardancy [23,35,36].

## 4. Conclusions

Within the scope of this study, the influence of different particle sizes in multi-component systems on the flame retardant as well as mechanical performance was investigated. Accordingly, material mixtures based on commercially available PBT, AlPi, and boehmite types were defined. The variation of particle sizes within the multi-component system was achieved using two chemically identical boehmite grades. Results of the investigation can be summarized, as follows:

- Partial substitution of AlPi by boehmite increases the tensile strength as well as the e-module and reduces the elongation at break. We conclude that this effect is caused by higher surface-volume ratios comparing of boehmite and AlPi particles, resulting in better polymer-particle bonding.
- PBT/AlPi/boehmite mixtures show synergism in LOI replacing 5 wt.% of AlPi with boehmite. We conclude that two effects cause the synergism. The efficiency of AlPi progressively decreases with concentration higher than 12–15 wt.% offering synergistic mixtures just by replacing less efficient AlPi by a second flame retardant. The clear outperforming of PBT/ AlPi only for adding a certain amount evidences that an additional synergistic interaction occurs.
- PBT/AlPi/boehmite mixtures containing larger as compared to smaller boehmite particles, increased the LOI value by 4%. We conclude that this effect is caused by an earlier water release of smaller boehmite particles changing the decomposition path, in particular during the beginning of pyrolysis. This leads to a quantitatively higher formation of highly flammable tetrahydrofuran due to hydrolysis.
- At higher heating rates, inertial effects of larger boehmite particles (B60) show a much greater shift in water release towards higher temperatures than smaller boehmite particles (200SM). Thus, an earlier water release of smaller boehmite particles in PBT/AlPi/boehmite mixtures lead to higher water concentrations being available at lower temperatures. We conclude that higher heating rates promote a higher THF production due to hydrolysis.
- The decomposition of PBT/AlPi/boehmite mixtures resulted in higher amount of residue. The residue amount increases with applying smaller boehmite particles, most probably due to the higher activity of the formed metal oxides catalyzing some carbonaceous charring. What is more, the morphology of fire residues strongly changed. Adding mixtures of AlPi and boehmite yields the formation of residual protective layers. We conclude that the combination of flame-retardant modes of action in the gas phase, flame inhibition due to AlPi, and the flame-retardant modes of action in the condensed phase, charring, and protective layer formation, causes the pronounced synergy.

## Figures and Tables

**Table 1:** Design of experiment—polymer/flame retardant recipe.

Symbol	PBT (wt.%)	Ratio AlPi/Boehmite	AlPi (Exolit 1240) (wt.%)	Boemite (AlOOH) B60; d_90_ < 5 μm (wt.%)	Boemite (AlOOH) 200SM; d_90_ < 0.6 μm (wt.%)
A1	100				
A2	90		10		
A3	85		15		
A4	80		20		
B1	80	3:1	15	5	
B2	80	1:1	10	10	
C1	80	3:1	15		5
C2	80	1:1	10		10

**Table 2:** Heating rates and test atmospheres thermogravimetric analysis (TGA) and TGA-fourier transformation infrared spectroscopy (TGA-FTIR) measurements—overview.

Symbol	Materials (wt.%)	TGA- Standard	TGA-High Speed	TGA & FTIR	Test Atmosphere
A1	PBT	20 K/min			N_2_, O_2_
-	AlPi (powder)	20 K/min			N_2_, O_2_
-	B60 (powder)	20 K/min	200, 500 K/min		N_2_, O_2_
-	200SM (powder)	20 K/min	200, 500 K/min		N_2_, O_2_
A2	PBT + 10% AlPi	20 K/min			N_2_, O_2_
A3	PBT + 15% AlPi	20 K/min			N_2_, O_2_
A4	PBT + 20% AlPi	20 K/min	200, 500 K/min		N_2_, O_2_
B1	PBT + 15% AlPi + 5% B60	20 K/min		5 K/min	N_2_, O_2_
C1	PBT + 15% AlPi + 5% 200SM	20 K/min		5 K/min	N_2_, O_2_

**Table 3:** Important key values of TGA measurements of all material mixtures under nitrogen and oxygen atmosphere.

		N_2_				O_2_			
	Material Compositions (wt.%)	*T*_95_ (°C)	*T*_50_ (°C)	Char Residue (%)	Char Residue Calc (%)	*T*_95_ (°C)	*T*_50_ (°C)	Char Residue (%)	Char Residue Calc. (%)
A1	PBT	380	412	≈6%	-	325	397	≈0%	-
-	AlPi (powder)	452	493	≈26%	-	319	408	≈46%	-
-	B60 (powder)	495	-	≈82%	-	495	-	≈82%	-
-	200SM (powder)	460	-	≈82%	-	460	-	≈82%	-
A4	PBT + 20%	370	412	≈11%	10%	364	401	≈9%	14%
B1	PBT + 15% + 5% B60	370	412	≈13%	13%	365	404	≈11%	16%
C1	PBT + 15% + 5% 200SM	370	412	≈17%	13%	354	401	≈13%	16%

**Table 4:** Overview of the characteristic molecules identified vs. literature values.

		Literature Values (cm^−^^1^)		
Molecule	Characteristic Wavelength(cm^−1^)	PBT/PBT + AlPi [12]	PBT [31]	PBT/PBT + AlPi [18]
1,3 butadien	908	908	905	908
therephthalic esters	1743, 1267, 1100	1743, 1267, 1100	1737, 1265, 1100	1743, 1267, 1100
benzoic acid	3578, 1758, 1178	3580, 1760, 1177		3580, 1760, 1177
CO	2110, 2181			
CO_2_	2450–2300, 668	2354, 2334, 667	3580. 1760, 669	2354, 667
tetrahydrofuran (THF)	2978, 1083	2980, 1083	2993, 2871, 1079	2980, 1083
1,4-butanediol			2982, 2987, 1072	
benzene		672		
diethylphosphinic acid	708, 850, 1018	1017, 853, 773, 650		3650, 850, 773
ethene		950		
